# T Cell Response to Infliximab in Exposed Patients: A Longitudinal Analysis

**DOI:** 10.3389/fimmu.2018.03113

**Published:** 2019-01-11

**Authors:** Sara Pratesi, Francesca Nencini, Francesca Grosso, Laura Dies, Susanna Bormioli, Daniele Cammelli, Enrico Maggi, Andrea Matucci, Alessandra Vultaggio, ABIRISK Consortium

**Affiliations:** ^1^Centre of Research DENOTHE, Department of Experimental and Clinical Medicine, University of Florence, Florence, Italy; ^2^Immunoallergology Unit, Careggi University-Hospital, Florence, Italy; ^3^Immunology Research Area, IRCSS Bambino Gesù Pediatric Hospital, Rome, Italy

**Keywords:** anti-drug antibodies, IL-10, immunogenicity, infliximab, IFX-specific T cells

## Abstract

This study aimed to evaluate the proportion of infliximab (IFX)-exposed patients exhibiting cellular response to the drug in a longitudinal way and to establish whether it is predictive for anti-drug antibodies (ADA) development. Seventeen patients suffering from immuno-mediated disorders were enrolled. Blood was sampled at baseline and before each of the first eight infusions of IFX. The proliferation of PBMCs to 15-mer peptides covering VH/VL frames of IFX was assessed as well as transcription factors and cytokines mRNA expression of memory T cells in IFX-stimulated PBMCs. The number of peptides recognized by T cells after four infusions was higher than those recognized by the same patients before treatment. IFX-stimulated PBMCs from more than 90% of patients were able to express the main regulators and adaptive cytokines of memory T cells. While IFN-γ mRNAs increased after the first infusion and declined during the subsequent ones, IL-10 mRNA was upregulated throughout the treatment. IL-10 was functionally active because its neutralization improved IFN-γ and IL-13 mRNA expression *in vitro*. The IL-10/IFN-γ ratio was shown to be lower in patients who developed ADAs solely at the early infusions. IL-10 production consistently preceded or paralleled the IFN-γ onset in ADA– patients, while it was not produced or followed IFN-γ onset in ADA+ patients. In conclusion, this study provides evidence that the majority of exposed patients undergo a cellular response to IFX with the upregulation of IL-10. The development of ADA is associated with the early impairment of IL-10 and low levels of the IL-10/IFN-γ ratio.

## Introduction

Infliximab (IFX) is a TNF-α blocker with known efficacy in several immune-mediated disorders, since it interferes effectively with inflammatory cascades (1, 2). IFX is a chimeric monoclonal antibody (mAb) composed of the constant region of human IgG1k and the murine VH and VL frames (3, 4). Due to its xenogenic epitopes, IFX is potentially immunogenic in a proportion of patients, leading to the onset of anti-drug antibodies (ADA) (5), a condition associated with loss of response (LOR) or hypersensitivity reactions (HRs) toward the drug (6–8). Our previous results indicated that IFX-specific T cells were detectable in the blood in 56% of ADA+ patients, especially when they experience HR (9). We also showed that IFX treatment usually leads to the development of IL-10- producing memory T cells in exposed patients, likely contributing to regulate drug immunogenicity (10).

This paper sought to evaluate, in a longitudinal manner, the development of a cellular response to IFX in treated patients, and its predictive role for ADA development. We showed that the great majority of 17 consecutive exposed patients developed drug sensitization, since IFX-stimulated PBMCs expressed mRNAs of main regulators and adaptive cytokines and recognized IFX peptides. However, the majority of exposed patients were also able to produce functionally active IL-10 able to regulate the production of cytokines from memory T cells. Lastly and more importantly, ADA+ patients showed higher IFN-γ and lower IL-10 than ADA– patients, thus suggesting their predictive role in ADA development.

## Materials and Methods

### Reagents

Forty six lyophilized 15 mer (overlapping 10 mer) peptides covering the VH and VL sequences of IFX (Primm, Milan, Italy) were dissolved in sterile water at 1 mg/ml. Low-endotoxin RPMI 1640 medium (VLE-RPMI 1640, Biochrom AG, Germany) was supplemented with 2 mM L-glutamine, 2 mM 2-mercaptoethanol, 100 U/ml penicillin and 100 μg/ml streptomycin, 1% non-essential amino acids, 1% sodium pyruvate (all from Sigma Chemical Co, Milan, Italy) (complete medium). Human serum AB was purchased from Euroclone (Milan, Italy). Phorbol 12-myristate 13-acetate (PMA) and ionomycin (I) were purchased from Sigma-Aldrich (Milan, Italy). Anti-MHC class II antibody and isotype control were purchased from Becton-Dickinson (Mountain View, CA). Anti-human IL-10 antibody, anti-human IL-10R alpha monoclonal antibody and their isotype controls were purchased from R&D Systems (Minneapolis, MN).

### Patients and Sampling

Blood were sampled from a cohort of 17 consecutive patients suffering from Inflammatory Bowel Diseases—IBD—(n°1), Vasculitis—VAS—(n°3), Rheumatoid Arthritis—RA—(n°5), Spondiloarthritis—SPA—(n°4), and Psoriasic Arthritis—PA—(n°4) who started treatment with IFX. Patients were infused with infliximab over a 2 h period at 0, 2, 6 weeks and every 8 weeks thereafter. The analysis was performed on consecutive samples taken at the time 0 (before treatment) and at the subsequent eight infusions (t1–t8). A control group of eight patients assayed before (t0) and after (t4) the switch from IFX originator to its biosimilar [CT-P13 (11)] was also analyzed for the response to IFX peptides. In order to minimize the interference with the drug for ADA evaluation, all samples were collected immediately before each infusion.

Clinical responses were determined by disease-specific scores on the day of blood sampling. Specifically, for the IBD patients, the Mayo Score Index and the Harvey-Bradshaw index score for ulcerative colitis and Crohn's disease were, respectively used (12, 13). For the RA patients, the delta Disease Activity Score (ΔDAS28) according to the EULAR response criteria was used (14). For the SpA patients, we used the Bath Ankylosing Spondylitis Disease Activity Score (BASDAI), and the response to treatment was defined according to the ASAS consensus statement for the use of TNF-α inhibitors in SpA (15). For the Vas patients, the clinical response was assessed using the Birmingham Vasculitis Activity Score version 3 (BVAS.v3) (16).

During IFX infusions four of 17 patients were co-treated with Oral corticosteroids (OCS), three with OCS plus Methotrexate (MTX), one with MTX alone, one with OCS plus others Disease modifying anti-rheumatic drugs (DMARDS) and one with salazopirina.

During the treatment, five patients developed ADA: one at t1, three at t4 and one at t5.

Six patients displayed negative outcomes: five developing LOR (three ADA– and two ADA+) and one ADA+ developing HR.

The patients' demographic, clinical and laboratory characteristics are summarized in Table 1. The study was approved by the local Ethics Committee (2012/0035982), and written informed consent was received from the participants before their inclusion in the study.

**Table 1:** Patients' characteristics.

	**Sex**	**Age**	**Disease**	**Disease duration (Years)**	**Concomitant therapy**	**IFX peptides proliferation**	**mRNA analysis**
Patient 1	F	78	RA	10	MIX + OCS	+	+
Patient 2	F	56	SPA	6	–	–	+
Patient 3	M	40	PA	8	–	+	+
Patient 4	M	56	VAS	4	ocs	+	+
Patient 5	M	65	RA	3	MTX	+	+
Patient 6	M	40	IBD	4	ocs	–	+
Patient 7	F	68	PA	5	–	+	+
Patient 8	M	26	PA	12	–	–	+
Patient 9	F	51	RA	5	MIX + OCS	+	+
Patient 10	F	28	PA	4	MIX + OCS	+	+
Patient 11	F	47	RA	10	DMARD + OCS	–	+
Patient 12	F	63	SPA	6	–	–	+

*F, female; M, male; RA, Rheumatoid Arthritis; SPA, Spondiloarthritis; PA, Psoriasic arthritis; VAS, vasculitis; IBD, Inflammatory Bowel Diseases; MTX, Methotrexate; OCS, Oral corticosteroids; DMARD, Disease modifying anti-rheumatic drugs*.

### Lymphocyte Proliferation Test (LTT)

Peripheral mononuclear cells (PBMCs) from patients were isolated with Ficoll-Paque, and 2 × 10^5^ cells were cultured for 5 days in complete medium and 5% heat-inactivated human serum AB in 96-well round-bottomed microwell plates with or without IFX (50 μg/ml) and IFX peptides (5 μg/well). Sixteen hours before harvesting, 0.5 μCi of Tritiated Thymidine (PerkinElmer, Boston, USA) was added to each well, and radionuclide uptake was measured by scintillation counting (MicroBeta TriLux PerkinElmer, Boston, USA). Mitogenic index (ratio between mean value of counts per minute—c.p.m.—of samples and medium alone, MI) ≥2 was considered positive as described (10).

For evaluation of mRNA expression, 1 × 10^6^ cells were cultured for 12 h in complete medium and 5% heat-inactivated human serum AB in 48-well round-bottomed microwell plates with or without IFX (50 μg/ml) (10).

### Real-Time PCR

RNA was extracted with the RNeasy mini kit (Qiagen, Milan, Italy), and cDNA was transcribed using a reverse transcription kit (Applied Biosystems, Warrington, UK) according to the manufacturer's instructions. The real-time PCR reactions were performed in multiple replicates and run on an ABI PRISM 7700 Sequence Detector (Applied Biosystems, Warrington, UK) with predesigned TaqMan Gene Expression Assays and reagents (Applied Biosystems, Warrington, UK), according to the manufacturer's instructions.

Regarding the longitudinal study, the procedures of RNA extraction and reverse transcription were performed immediately after the sampling, while the cDNA amplification of all samples were performed simultaneously. mRNA onset (ratio between the value of cytokine gene expression produced in response to the IFX and the value of cytokine gene expression produced in response to medium alone) ≥2 was considered positive.

### ELISA Assays

Supernatants obtained from LTT with peptides were assayed for IFN-γ, IL-13, IL-17A, and IL-10 with commercially available ELISA kits (R&D Systems, Minneapolis, MN), according to the manufacturer's instructions.

The ADA status of patients was evaluated by using a commercially available bridging ELISA kit (Promonitor, Progenika Biopharma, Bizkaia, Spain), according to the manufacturer's instructions.

### Statistical Analysis

The results are presented as mean values ± SEM. The statistical analyses were performed using Student paired *t*-test and Wilcoxon signed-rank test. *P* < 0.05 were considered significant.

## Results

### Increased Proportion of Circulating T Cells Specific for Drug Peptides During IFX Treatment

We designed a 1-year longitudinal study on a panel of sequential outpatients suffering from immuno-mediated disorders and undergoing IFX treatment at the Clinical Immunology Unit of Careggi-University Hospital, Florence (Italy).

**Figure 1:**
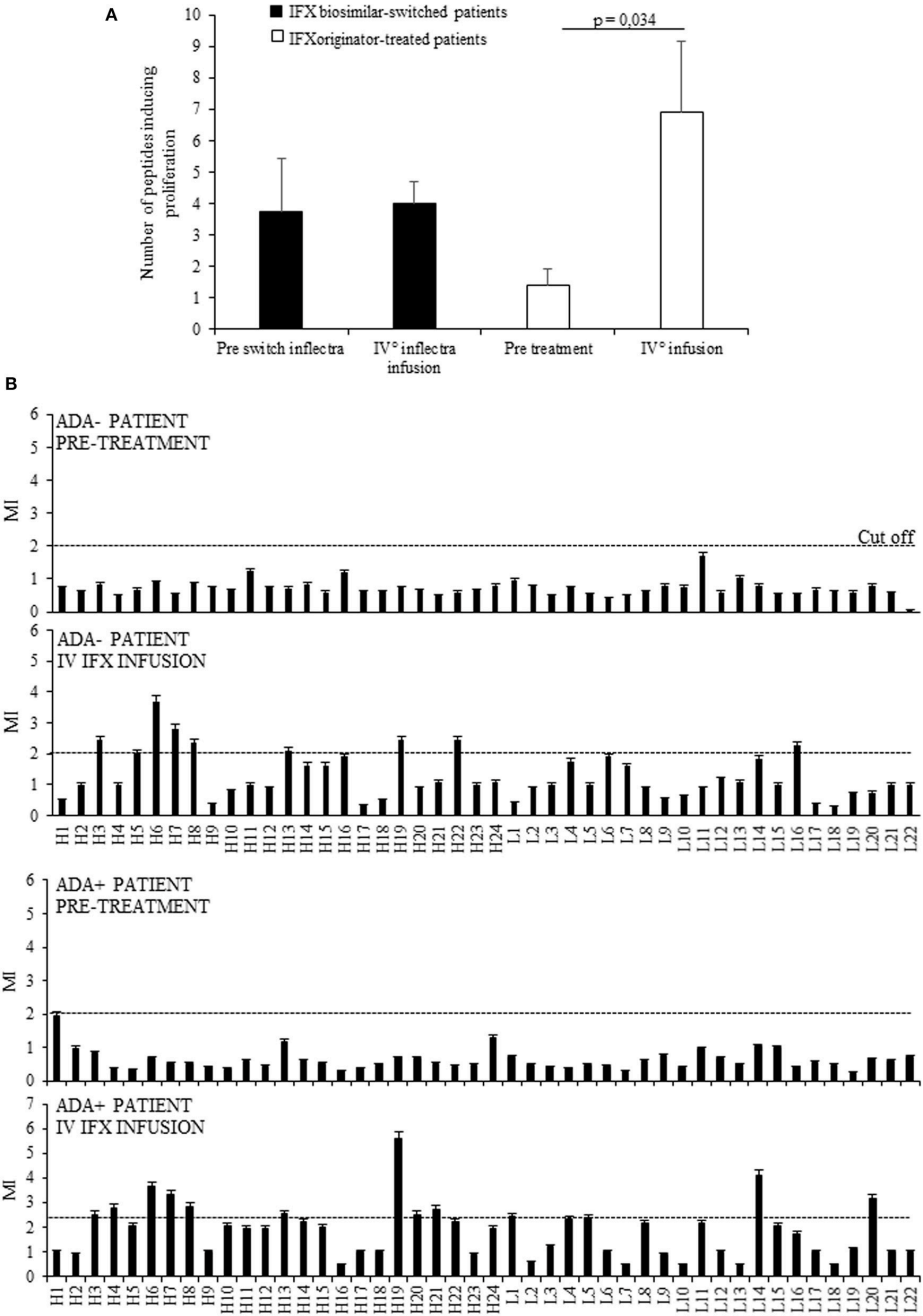
Evaluation of T cell proliferation to IFX peptides in patients before and after drug infusion. **(A)** Mean number of peptides inducing proliferation in 11 patients analyzed at t0 and at t4 (white columns) and in eight patients assayed before (t0) and after (t4) the switch from IFX originator (Remicade) to biosimilar (Inflectra) (black columns). **(B)** Proliferative response to IFX peptides in one ADA– patient and one ADA+ patient before and after the treatment. Data are expressed as mean values ± SEM. The statistical analysis was performed using Student paired *t*-test. MI, mitogenic index; H, V region of heavy chain; L, V region of light chain.

Initially we assessed the proliferative response of PBMCs from 11 selected patients to the drug and to 46 15 mer-peptides (10 mer overlapping) covering the VH and VL frames of IFX, at t0 (before the treatment), and t4 (before the fifth infusion). This analysis provided evidence that the number of recognized peptides was shown to be significantly higher at t4 (n°91) than those recognized at t0 (n°18). By contrast, the number of recognized peptides was unchanged in PBMCs from a control group of patients, represented by eight patients assayed before (t0) and after (t4) the switch from IFX originator to its biosimilar (Figure 1A). The response to each peptide was MHC class II-restricted since the proliferative response of PBMCs plus peptide was abrogated by co-culturing with anti-MHC Class II, but not control isotype, mAbs (data not shown).

Furthermore, while no PBMCs from selected patients displayed proliferation to the entire drug *in vitro*, the peptide recognition was increased during the treatment in nine (81.8%) patients, four ADA+ (100%), and five ADA (71.4%), thus suggesting that the majority were sensitized to IFX. A representative graph of IFX peptides proliferation in ADA– and ADA+ patients was shown in Figure 1B.

### Increased mRNA Expression of Transcription Factors, Adaptive, and Regulatory Cytokines in Drug-Stimulated PBMCs During IFX Therapy

To establish the proportion of exposed patients undergoing IFX-specific T cell response, we analyzed longitudinally the mRNA expression of main regulator transcription factors of Th1/Th2/Th17 cell subsets (T-bet, GATA3, RORC) and of their related cytokines (IFN-γ, IL-13, IL-17A) in drug-stimulated PBMCs. The study was performed on 17 (the previous 11 plus another 6) patients, sampled before the start of treatment (t0) and before each infusion (t1–t5 and t8).

**Figure 2:**
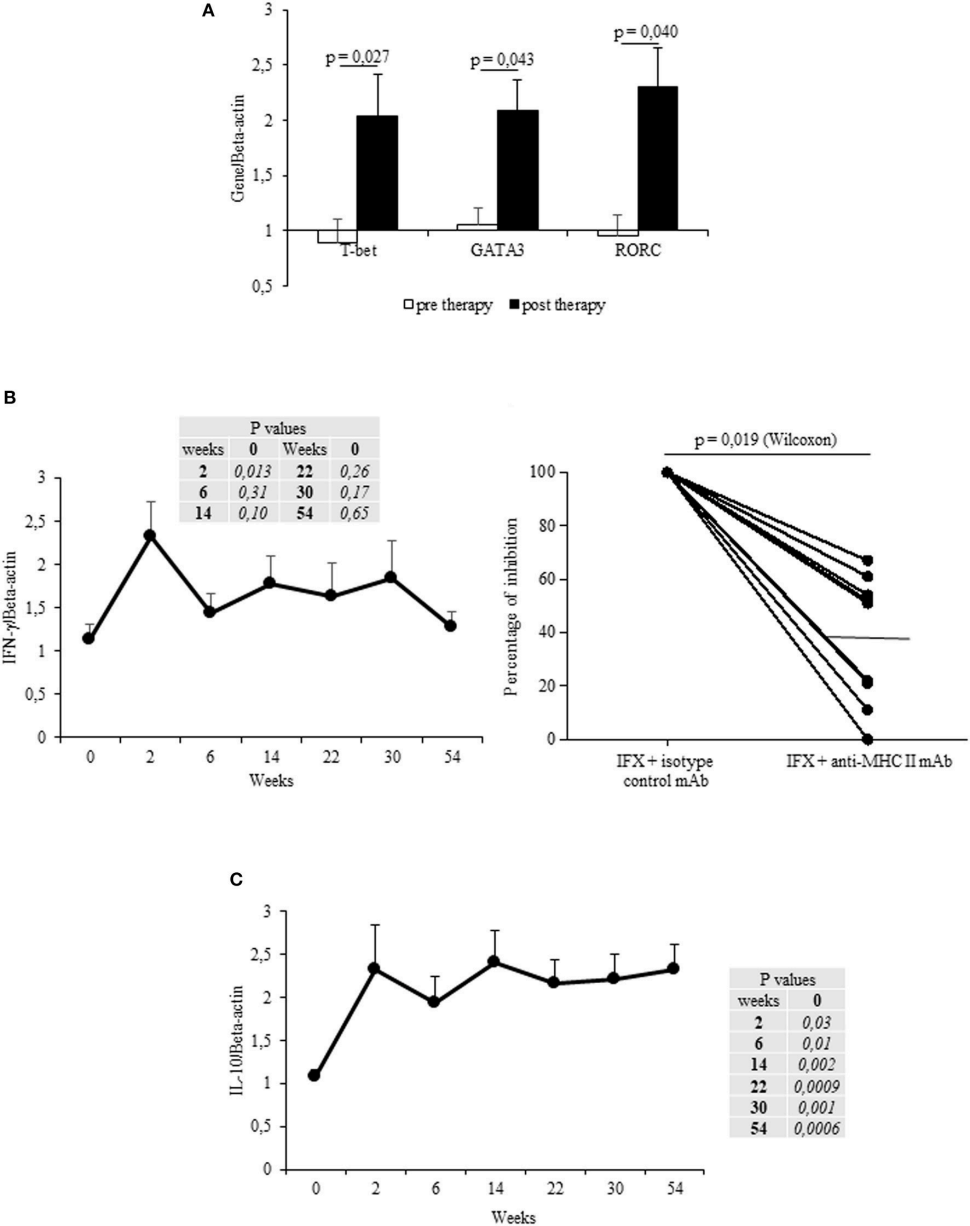
Longitudinal evaluation of transcription factors and cytokines in IFX-stimulated PBMC from exposed patients. **(A)** mRNA expression of main regulator transcription factors (T-bet, GATA3, RORC) in drug-stimulated PBMCs before and after therapy (evaluated at t1). **(B)** IFN-γ mRNA expression in drug-stimulated PBMCs at different time points (left panel). Percentage of inhibition of IFN-γ mRNA expression in PBMCs cultured with IFX in the presence of anti-MHC II, or control isotype, mAbs (10 μg/ml) for 12 h (right panel). **(C)** IL-10 mRNA expression in drug-stimulated PBMCs at different time points. Data are expressed as mean values ± SEM. The statistical analysis was performed using Student paired *t*-test **(A–C)** and Wilcoxon signed-rank Test **(B)**.

The mean values (±SEM) of T-bet, GATA3 and RORC mRNA expression in IFX-cultured PBMCs from all patients were increased after therapy vs. the baseline (Figure 2A). T-bet mRNA expression increased in 13/17 patients (76%) upon beginning IFX in comparison to the baseline, whereas GATA3 and RORC mRNAs were both upregulated in 10/17 (58.8%) and 12/17 (70.6%) patients, respectively.

By analyzing the cytokines mRNAs expression in IFX-cultured PBMCs from all patients, a significant (*p* = 0.013) increase of IFN-γ (mean values ± SEM) was observed after the first infusion, though it declined at subsequent time points (t2–t5, t8) (Figure 2B, left panel). By contrast, IL-13 and IL-17 mRNAs did not significantly increase (data not shown). The increase in IFN-γ was observed in 15 patients, 8 of them displaying an early increase (at t1), while IL-13- or IL-17 mRNA expression increased in six (35.3%) patients during the entire treatment. Notably, the main regulator (T-bet) and cytokine (IFN-γ) mRNA expression in IFX-stimulated PBMCs (sampled at t1) were MHC Class II restricted (Figure 2B right panel and data not shown).

Since we recently showed that IFX treatment expands a proportion of memory T cells producing functionally active IL-10 (10), in this study we longitudinally evaluated the production of this molecule, focusing on its regulatory activity to adaptive cytokines.

By analyzing the kinetics of IL-10 mRNAs in IFX-cultured PBMCs from the selected patients, we found that IL-10 mRNA was increased (vs. t0) from t1 to t8 (Figure 2C). Cumulatively, the IL-10 mRNAs were shown to have increased in the PBMCs from 15 patients (88.2%). Importantly, while PBMCs from ADA– patients expressed IL-10 mRNAs at all time points, only three out of the five patients that developed ADAs showed IL-10 mRNAs.

### PBMCs From Patients Developing ADAs Showed Low Levels of Drug-Inducible IL-10 During the Treatment

Since five of the selected patients developed ADAs during the treatment, we subsequently analyzed the kinetics of IL-10 and IFN-γ mRNAs in ADA+ and ADA– patients.

**Figure 3:**
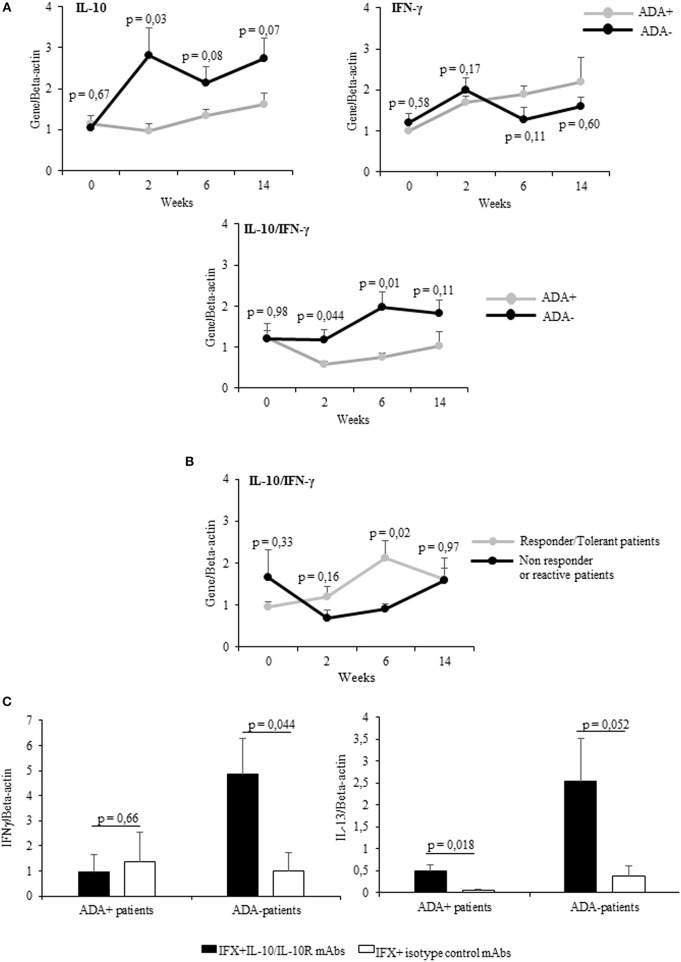
PBMCs from patients developing ADAs showed low levels of drug-inducible IL-10. **(A)** mRNA expression of IL-10, IFN-γ (upper panel), and IL-10/IFN-γ ratio (lower panel) longitudinally evaluated in drug-stimulated PBMCs from ADA+ (*n* = 5) and ADA– (*n* = 12) patients. **(B)** IL-10/IFN-γ ratio longitudinally evaluated in drug-stimulated PBMCs from patients with different clinical outcomes. **(C)** IFN-γ (left panel) and IL-13 (right panel) mRNA expression in PBMCs obtained from ADA+ patients and ADA– patients cultured for 12 h with IFX in the presence of IL10/IL10R, or control isotype, mAbs (10 μg/ml). Data are expressed as mean values ± SEM. The statistical analysis was performed using Student paired *t*-test.

Cumulatively the mean values (±SEM) of IL-10 mRNA expression detected at different time points were higher (significant at t1) in ADA– patients than in ADA+ patients (Figure 3A upper panel), while IFN-γ values were shown to be higher (though not significant) in ADA+ patients than in ADA– patients (Figure 3A upper panel). By analyzing the kinetics of cytokines in the two groups of patients, the IL-10/IFN-γ ratio of mRNA values was shown to be significantly lower in ADA+ patients than in ADA– patients at t1 and t2 (*p* = 0.044 and *p* = 0.01, respectively) (Figure 3A lower panel).

Taking into account the clinical outcome, the IL10/IFN-γ ratio was higher in patients with good clinical response than in patients with negative clinical outcome. This difference reached the statistical significance at t2 (Figure 3B). To establish whether IL-10 was active on adaptive cytokines produced by ADA+ patients and ADA– patients in a similar manner, we assessed the expression of IFN-γ mRNA in IFX-driven PBMCs (sampled at t2) from 5 ADA– patients and 5 ADA+ patients cultured in the presence of anti-IL-10/IL-10R or control isotype mAbs. Whereas, IFN-γ increased significantly (*p* = 0.044) only in PBMCs from ADA– patients upon IL-10 neutralization (Figure 3C), IL-13 mRNA increased in PBMCs from ADA– (*p* = 0.052) and ADA+ patients (*p* = 0.018).

**Figure 4:**
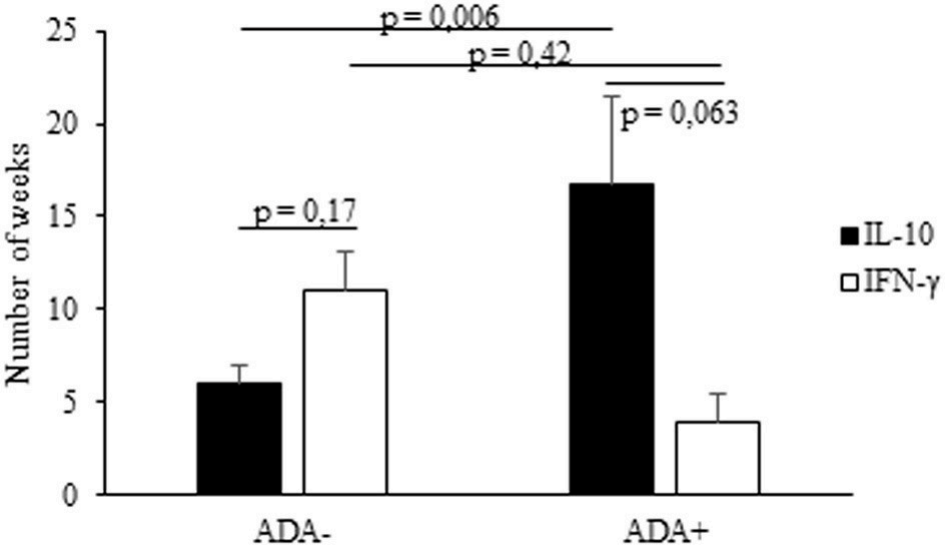
Timing of IL-10 and IFN-γ mRNA expression in PBMCs from IFX-treated patients. The onset (expressed as number of weeks) of IL-10 and IFN-γ mRNA upregulation in ADA+ and ADA– patients. Data are expressed as mean values ± SEM. The statistical analysis was performed using Student paired *t*-test.

Lastly, we analyzed the timing of the IFX-induced IL-10 and IFN-γ mRNA upregulation in PBMCs from the selected patients. The drug-driven increase of IL-10 mRNA preceded or paralleled that of IFN-γ in all ADA– patients, while that of IFN-γ preceded that of IL-10 in four of five ADA+ patients (Table 2). The timing (mean values ± SEM) of IL-10 mRNA onset was 16.7 ± 2.07 in ADA+ and 5.7± 1.5 weeks (*p* = 0.006) in ADA– patients, while that of IFN-γ mRNA onset was 4.0 ± 1.03 in ADA+ and 11.1 ± 4.79 in ADA– (*p* = 0.42) patients (Figure 4).

**Table 2:** Timing onset of TFs and citokines mRNA expression, ADA, and clinical outcome in IFX-treated patients.

	**TFsRNN^**°**^**	**IF*-y* mRNA^**°**^**	**IL-10 mRNA^**°**^**	**ADA onset^**°**^**	**Clinical outcome onset^**°**^**
**ADA+ PATIENTS (*****n*** **= S)**					
Patient 1	6	U	U	2	30 (LOR)
Patient 2	6	2	14	22	30 (HR)
Patient 3	2	6	u	22	14 (LOR)
Patient 4	2	2	22	22	- (Tolerant)
Patient 5	ND	6	14	30	- (Tolerant)
Mean ± SEM	4 ± 1	4 ± 1	16.7 ± 2.1^*^	19.6 ± 4.7^**^	24.7 ± 5.3^***^
**ADA– PATIENTS (*****n*** **= 12)**					
Mean ± SEM	14.6 ± 5.5	11.1 ± 4.8	5.7 ± 1.5	–	–

*HR, hypersensitivity reaction; LOR, loss of response; ND, not done; TFs, Transcription factors; U, undetectable*.

* *p = 0.006 IL-10 mRNA in ADA+ vs. ADA– patients*,

** *p = 0.001 ADA onset vs. IFN-y and TFs mRNA in ADA+ patients*.

*** *p = 0.007 Clinical outcome onset vs. IFN-y and TFs mRNA in ADA+ patients*.

° *Reported data are expressed in weeks*.

The drug-induced IFN-γ mRNAs detected in cultures from ADA+ patients also preceded the timing of ADA onset, the mean values (±SEM) being 4.0 ± 1.03 vs. 19.6 ± 4.7 weeks (*p* = 0.001), while that of IL-10 mRNAs did not (16.7 ± 2.07 vs. 19.6 ± 4.7 weeks, *p* = 0.22).

## Discussion

This longitudinal study firstly aimed to evaluate the proportion of exposed patients showing cellular sensitization to IFX (regardless of their ADA status) by analyzing the PBMCs response to peptides covering the VH and VL frames of the drug and the mRNA expression of main regulators and adaptive cytokines of memory Th cells in IFX-stimulated PBMCs. The crucial data of the paper is that the great majority of treated patients develop cellular response to the drug, which is also detectable after the first infusions. Even though rare peptides induced a proliferative response in PBMCs from some unexposed patients, likely due to cross-reactive epitopes, the longitudinal analysis indicated that the number of recognized peptides increased in over 80% of patients after the beginning of treatment. Accordingly, in the great majority of selected patients (88.2 and 76%, respectively) we observed an increase in IFN-γ and T-bet mRNA expression. Importantly, the MHC class II restriction of the peptide proliferation and the response of transcription factors and related cytokines confirmed the presence of memory T cells as the result of drug sensitization in the great majority of exposed patients. Regarding the time of onset of immune response against the drug, our data show that the mRNA expression for adaptive cytokines was detectable very early (usually after the first infusion) in the five ADA+ patients, always preceding ADA onset. Moreover, we did not observe any significant differences between patients with different diseases (data not shown).

The detection of a drug-specific cellular response in the great majority of treated patients raises the question as to why only some of them develop ADA. Several mechanisms are likely responsible for blocking ADA development. Salinas and colleagues provided evidence that TNF-α blocks the induction of T-cell dependent humoral response by interfering with the germinal center dependent B cell maturation (17). Others have shown that TNF-α blockers trigger transmembrane (tm) TNF-α expressing cells by outside-to-inside (reverse) signaling which leads to drug-mediated G0/G1 cell cycle arrest and apoptosis (5, 7). Finally, we and others have shown that TNF-α neutralization increased the development of memory CD4+ T cells producing IL-10, exhibiting an overall regulatory gene profile and showing enhanced suppressive function (10, 18).

For this reason, we longitudinally analyzed the IL-10 mRNA kinetics in exposed patients. As expected, we found that it was upregulated in the great majority of them, usually starting very early. These data were also confirmed by the presence of IL-10 in some peptide-stimulated culture supernatants for the majority of treated patients (unpublished data). In addition, while IFN-γ peaked after the first drug administration and declined to baseline level at subsequent time points, high levels of IL-10 mRNA were maintained during the treatment in the large majority of patients. In fact, we have recently shown that IFX-driven IL-10 was functionally active, since its neutralization resulted in upregulation of adaptive cytokines (10). In addition, we have also provided evidence that IFX-specific T-cell clones producing IL-10 exerted an inhibitory activity on the response to IFX of autologous T effector cells (10).

Lastly, taking advantage of the fact that five of the selected patients developed ADA during the treatment, we analyzed the kinetics of IL-10 and IFN-γ mRNAs in ADA+ patients and ADA– patients in order to evaluate their potential predictive role for ADA development. While in the former group IFN-γ mRNA levels were consistently higher than those of IL-10, this latter overrode IFN-γ in ADA– patients. Importantly, the IL-10/IFN-γ ratio was shown to be significantly higher in ADA– patients than in ADA+ patients, at least at the early infusions. If this result will be confirmed in a larger number of patients longitudinally analyzed, IL-10/IFN-γ might be considered a potential predictive marker of ADA development.

A further confirmatory result is the different timing of cytokine expression in ADA+ patients and ADA– patients. Indeed, we found that IL-10 consistently preceded or paralleled the IFN-γ onset in ADA– patients, while it followed IFN-γ (or was not produced) in ADA+ patients. On the whole, these results suggest a different ability to produce IL-10 in response to IFX stimulation in ADA+ patients and ADA– patients, because we found that the IL-10 neutralization induced the IFN-γ upregulation in cultures from ADA– patients but not ADA+ patients. These data agree with a previous study at the genetic level showing that ADA onset to Adalimumab (another TNF-α blocker) in RA patients was significantly associated with IL-10 gene polymorphism (19).

Even though the limitation of this study lies in the very small number of patients, mainly in those testing ADA+, it suggests a predictive role of low levels of IL-10 on the development of ADA. The impaired ability to produce IL-10 in response to IFX in ADA+ patients was indirectly confirmed from the data on PBMCs from five ADA+ patients sampled immediately before the severe post-infusion reaction to the drug. In these cultures, IFX stimulated no IL-10 mRNA expression, while, conversely, several IFX peptides induced IFN-γ, but not IL-10, in the supernatants of cultures performed after the reaction (Vultaggio A., unpublished data).

Overall, the results of this study strongly suggest that the great majority of exposed patients undergo cellular adaptive response to IFX during the early infusions. The subsequent ADA development occurring in a proportion of exposed patients is likely associated with the fine balance between the IFX-driven production of regulatory (IL-10) and adaptive (IFN-γ) cytokines.

## Ethics Statement

All procedures performed in studies involving human participants were in accordance with the ethical standards of the Local Ethical Committee (Ethical Committee Area Vasta Centro Firenze, Azienda Ospedaliera Universitaria Careggi, Largo Brambilla 3, 50134 Florence, Italy- protocol number 2012/0035982) and with the 1964 Helsinki declaration and its later amendments or comparable ethical standards.

## Author Contributions

AV and EM conceived the study, designed the experiments, interpreted the results, and drafted the manuscript. AM participated in study design and interpretation and in drafting of the manuscript. SP and FN performed experiments and participated in acquisition and interpretation of the data. FG, LD, SB, and DC participated in study enrolling patients.

### Conflict of Interest Statement

The authors declare that the research was conducted in the absence of any commercial or financial relationships that could be construed as a potential conflict of interest.
